# Mosaic Trisomy 18 in a Five-Month-Old Infant

**DOI:** 10.1155/2013/929861

**Published:** 2013-05-27

**Authors:** Ana Laura Fitas, Mafalda Paiva, Ana Isabel Cordeiro, Luís Nunes, Gonçalo Cordeiro-Ferreira

**Affiliations:** ^1^Área de Pediatria Médica, Hospital de Dona Estefânia, Centro Hospitalar de Lisboa Central, EPE, Rua Jacinta Marto, 1169-045 Lisboa, Portugal; ^2^Serviço de Genética, Hospital de Dona Estefânia, Centro Hospitalar de Lisboa Central, EPE, Rua Jacinta Marto, 1169-045 Lisboa, Portugal

## Abstract

Individuals with mosaic trisomy 18, only approximately 5% of all trisomy 18 cases, carry both a trisomy 18 and an euploid cell line. Their clinical findings are highly variable, from the absence of dysmorphic features to the complete trisomy 18 syndrome. A five-month-old daughter of a 38-year-old mother, with vomiting and feeding problems, was referred to our department. She was undernourished and had axial hypotony and developmental delay, an irregular pattern of hypopigmentation on the right side of the abdomen, and moderate sagittal body asymmetry with left-side muscular hemihypotrophy. Mild craniofacial dysmorphy included dolichocephaly, frontal bossing, prominent *occiput*, long downslanting palpebral fissures, hypertelorism, and retrognathia. A complex heart defect with atrial and ventricular septal defects, pulmonary artery stenosis, and bicuspid aortic valve was identified. Cytogenetic analysis revealed mosaic trisomy 18 with trisomy in 90% of peripheral lymphocytes and 17% of skin fibroblasts. This case adds to our knowledge of the phenotypic spectrum and the natural history of mosaic trisomy 18 by adding a dysmorphic feature and a cardiac abnormality that, to the best of our knowledge, had not been previously described.

## 1. Introduction

Mosaicism is the presence of more than one cell line in the same individual, and it occurs in approximately 5% of trisomy 18 cases [1]. These individuals carry both a trisomy 18 and an euploid cell line. Their clinical findings are highly variable in a phenotypic spectrum that spans from the absence of dysmorphic features with normal intelligence to the complete trisomy 18 syndrome [2]. Edwards syndrome, the complete trisomy 18 phenotype, affects approximately 1 in 6,000 live births, of which only 10% survive beyond the first year of life [3]. It is characterized by multiple congenital anomalies, feeding difficulties, and severe psychomotor, and growth retardation. Some distinctive features are microphthalmia, micrognathia, clenched fingers, and rocker-bottom foot with prominent calcaneus. As these characteristics may be totally absent in mosaics, a high level of suspicion for the possibility of a mosaic cytogenetic abnormality is required. 

A 5-month-old girl with mosaic trisomy 18 is reported. This case adds to our knowledge of the spectrum and natural history of mosaic trisomy 18, for which case reports are a major contribution. It adds a dysmorphic feature and cardiac abnormality that, to the best of our knowledge, have not been previously described. 

## 2. Case Presentation

**Figure 1:**
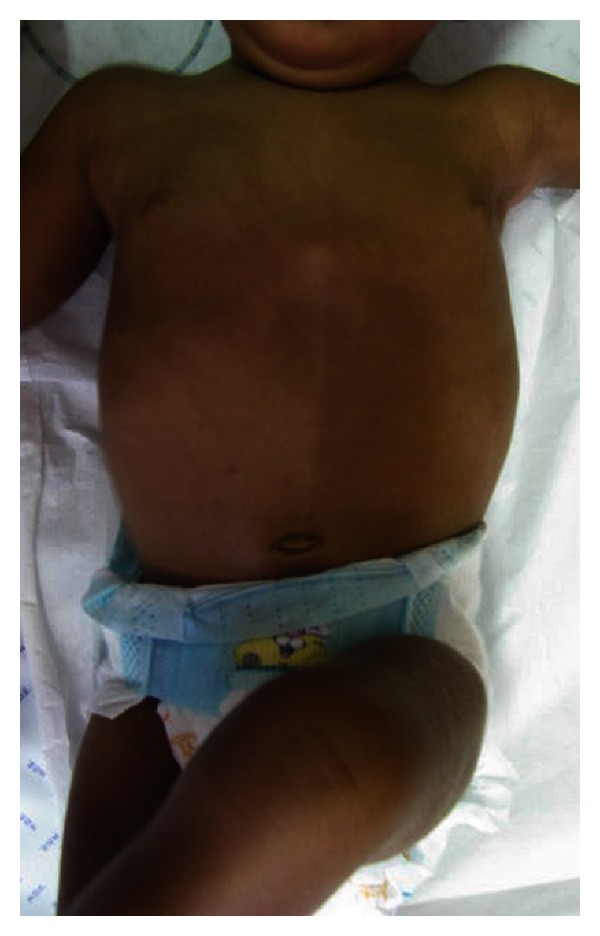
Irregular pattern of hypopigmentation area on the right side of the abdomen, not following the Blaschko lines.

A female fifth child of a 38-year-old mother was born in Cape Verde islands at the 41st week of an uncomplicated gestation, via lower segment caesarean section because of nonprogressing labor. Her birth weight was 2650 g, length of 46 cm, both adequate to gestational age; no information was available on the head circumference. No complications were referred in the neonatal period. Recurrent upper respiratory infections and laborious breathing began to occur from the second month of life, associated with frequent vomiting and feeding problems. She was transferred to our hospital at the age of 5 months. On admission she was undernourished, with weight 3850 g and length 57 cm (both below the 3rd centile). A IV/VI grade systolic murmur was present. On neurological examination she had axial hypotony, poor cephalic control, and absence of protection reflexes and did not attempt to reach for objects. She had an irregular pattern of hypopigmentation on the right side of the abdomen, not following the Blaschko lines (Figure 1), and a moderate sagittal body asymmetry with left-side muscular hemihypotrophy, particularly evident on the back and lower limbs. She also had rocker-bottom feet.

**Figure 2:**
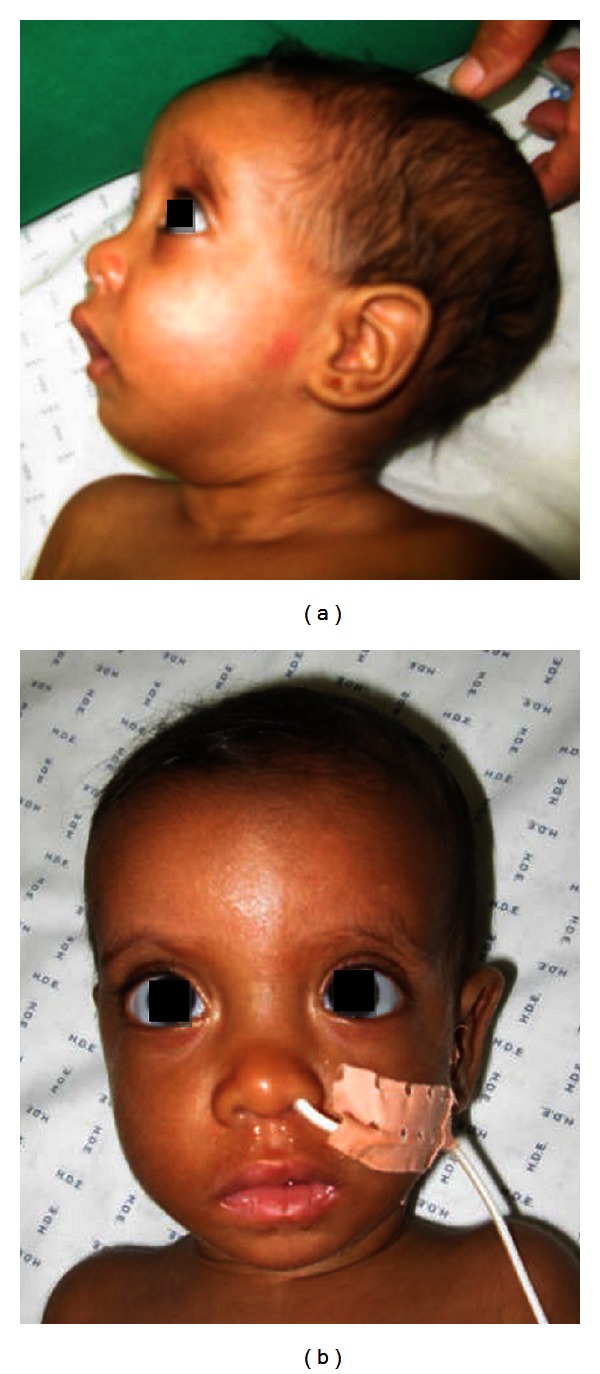
Lateral (a) and frontal (b) view of face and neck of the patient.

Mild craniofacial dysmorphy was present including dolichocephaly, narrow bifrontal diameter, frontal bossing, prominent *occiput*, low set ears and retrognathia (Figures 2(a) and 2(b)), long downslanting palpebral fissures and hypertelorism (Figure 2(b)), and high arched palate.

No abnormalities were found in the laboratory workup for common causes of failure to thrive with recurrent upper respiratory infections, frequent vomiting, and feeding problems, including immunoglobulin levels, sweat test, thyroid hormones, ant-itransglutaminase antibodies, and iron metabolism. No metabolic disease was identified on the Guthrie chart. Electroencephalogram and brain magnetic resonance imaging were normal.

Echocardiography showed complex heart defect with subaortic perimembranous ventricular septal defect, *ostium secundum* atrial septal defect, pulmonary artery stenosis, and bicuspid aortic valve. Overall ventricular function was normal.

Cytogenetic investigation of peripheral lymphocytes revealed mosaic trisomy 18 (47, XX + 18[26]/46, XX[3]), with approximately 90% of trisomic cells (74–96%, CI 95%). This evaluation was further complemented with cytogenetic investigation of skin fibroblasts, which revealed mosaic trisomy 18 (47, XX + 18[5]/46, XX[24]), in approximately 17% of trisomic cells (8–35%, CI 95%).

The admission to the ward and subsequent close outpatient care followup were lengthy. Intervention targeted mainly nutritional and developmental rehabilitation therapy. Feeding difficulties with vomiting and regurgitation were a major issue of concern, requiring enteric nutrition through nasogastric intubation and eventually percutaneous gastrostomy. The overall outcome was good, with weight gain and acquisition of developmental landmarks gradually approaching the expected profile for her age.

## 3. Discussion 

There are 35 reported cases of mosaic trisomy 18, of which 33 were included on a review in a recent publication [4] and two were reported since then [5]. The age and clinical circumstances at diagnosis were considerably variable, with 15 cases being diagnosed in adulthood, 11 cases between two and 18 years of age, and only nine cases under the age of two years, of which only three were diagnosed in the first year of life [4, 5]. One of them was 10 months old, presenting with failure to thrive, developmental delay, and dysmorphic features. He was the second of a pair of siblings with mosaic trisomy 18 [6]. The other two patients were both diagnosed in the neonatal period: one of them had multiple congenital anomalies and was initially diagnosed as complete trisomy 18; the other was identified on the laboratorial workup for intrauterine growth restriction [5]. 

In none of these three cases were hemihypotrophy and abnormal skin pigmentation reported in the first year of life. Hemihypotrophy and skin areas with abnormal pigmentation, either following or not the lines of Blaschko, are often found combined in chromosomal mosaicism [7] and should raise the clinical suspicion of mosaicism.

To the best of our knowledge, this case also adds previously unreported features to the phenotype, namely, the finding of long palpebral fissures, opposed to the more frequent event of narrow and short palpebral fissures, and the structural heart defect with bicuspid aortic valve, none of them described neither in the review published by Tucker [4] nor in the more recent cases by Banka et al. [5]. 

Failure to thrive and developmental delay were probably related to both the chromosomal abnormality and the consequence of the feeding difficulties, also reported by Banka et al. [5]. The excellent neurodevelopment and growth recovery after nutritional rehabilitation strengthen the role of malnutrition in the clinical picture. 

In the current clinical case, cytogenetic investigation was performed on the investigation of an infant with failure to thrive, developmental delay, congenital heart defect, and minor dysmorphic features. Maternal age was also considered as a positive argument to perform cytogenetic investigation. Although etiologic factors for mosaic trisomy 18 are still unclear, it is well recognized that advanced maternal age is an important factor that increases the risk of chromosomal nondisjunction [8].

The patient achieved a relatively benign phenotype, with good *catch-up* development, normal heart function without requiring surgical intervention, and relatively mild dysmorphic features in spite of a high percentage of trisomy 18 cells in peripheral lymphocytes (90%). This reinforces that there is no correlation between the individual's phenotype and the percentage of trisomic cells, both in the peripheral lymphocytes and in the skin fibroblasts [4]. The only relevant clinical correlate finding on the review was that most of the patients with normal karyotype on the majority of examined lymphocytes had normal intelligence, while most of the patients with predominant trisomic karyotype on the examined lymphocytes had some degree of delay or mental retardation [4]. Only continued followup will clarify this aspect in this patient.

This case also illustrates that there is no predictable relation between the percentage of trisomic cells in the peripheral leukocytes (90%) and the skin fibroblasts (17%). This discrepancy was reported in 48% of the cases for which both results were available [4, 5]. Even with a high percentage of trisomy 18 cells in a peripheral leukocyte sample, it is not currently possible to predict the percentage of trisomy 18 cells neither in the skin fibroblasts nor in the brain, gonads, or other key organs [4].
